# Matched molecular pair-based data sets for computer-aided medicinal chemistry

**DOI:** 10.12688/f1000research.3-36.v2

**Published:** 2014-02-21

**Authors:** Ye Hu, Antonio de la Vega de León, Bijun Zhang, Jürgen Bajorath

**Affiliations:** 1 Department of Life Science Informatics,B-IT, LIMES Program Unit Chemical Biology and Medicinal Chemistry, Rheinische Friedrich-Wilhelms-Universität, Bonn, D-53113, Germany

## Abstract

Matched molecular pairs (MMPs) are widely used in medicinal chemistry to study changes in compound properties including biological activity, which are associated with well-defined structural modifications. Herein we describe up-to-date versions of three MMP-based data sets that have originated from in-house research projects. These data sets include activity cliffs, structure-activity relationship (SAR) transfer series, and second generation MMPs based upon retrosynthetic rules. The data sets have in common that they have been derived from compounds included in the ChEMBL database (release 17) for which high-confidence activity data are available. Thus, the activity data associated with MMP-based activity cliffs, SAR transfer series, and retrosynthetic MMPs cover the entire spectrum of current pharmaceutical targets. Our data sets are made freely available to the scientific community.

## Introduction

The matched molecular pair (MMP) concept is widely applied in medicinal chemistry ^1– 4^. An MMP is defined as a pair of compounds that are only distinguished by a structural modification at a single site ^1^, i.e., the exchange of a substructure, termed a chemical transformation ^5^. MMPs are attractive tools for computational analysis because they can be algorithmically generated and they make it possible to associate defined structural modifications at the level of compound pairs with chemical property changes, including biological activity ^2– 4^. MMPs are usually chemically intuitive and easily accessible, which helps to bridge the gap between computational analysis and the practice of medicinal chemistry.

In the context of different studies, we have systematically generated MMPs through the mining of publicly available compound activity data. All possible MMPs have been derived from compounds active against currently available pharmaceutical targets. Then, MMPs have been used to explore structure-activity relationships (SARs) on a large-scale and from different viewpoints.

In a previous data article, we have reported and made publicly available a number of different data sets and computational tools developed in our laboratory ^6^. Here we describe three recently developed MMP-based data structures, which should be of interest for SAR analysis and compound design, and we also provide up-to-date versions of the corresponding data sets. It is anticipated that these data sets will be helpful as a resource for computer-aided medicinal chemistry applications. The data sets include MMP-based activity cliffs (i.e., MMP-cliffs), SAR transfer series, and MMPs derived on the basis of retrosynthetic fragmentation rules and were derived from all bioactive compounds currently available in the ChEMBL database (release 17) ^7, 8^. Only high-confidence activity data (as specified below) were considered. MMP-cliffs, SAR transfer series, and retrosynthetic MMPs provide comprehensive sources of SAR information. In addition, retrosynthetic MMPs are thought to increase the utility of computational MMP analysis for practical chemistry efforts because these second generation MMPs consider reaction information during molecular fragmentation, which sets them apart from standard MMPs originating from systematic fragmentation of all possible exocyclic single bonds in a molecule (as detailed below).

## Materials and methods

### Concepts

**Figure 1.**
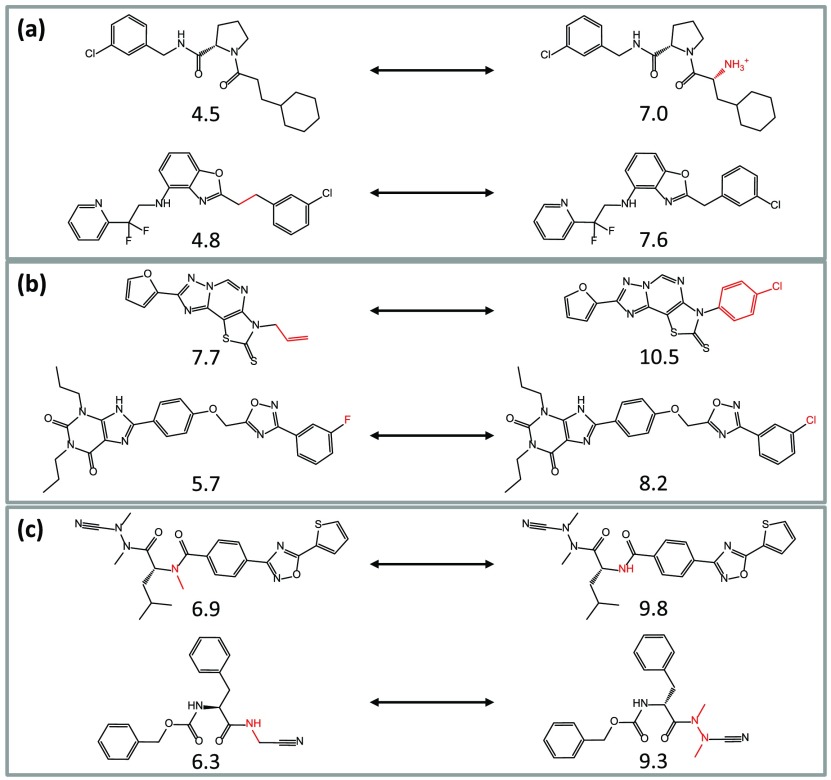
MMP-cliffs. Six representative MMP-cliffs for three targets belonging to different target families are shown; ( **a**) muscarinic acetylcholine receptor M3, ( **b**) serine/threonine-protein kinase c-TAK1, ( **c**) matrix metalloproteinase-2. The pK _i_ value of each compound is provided and the structural differences between cliff-forming compounds are highlighted in red.

**Figure 2.**
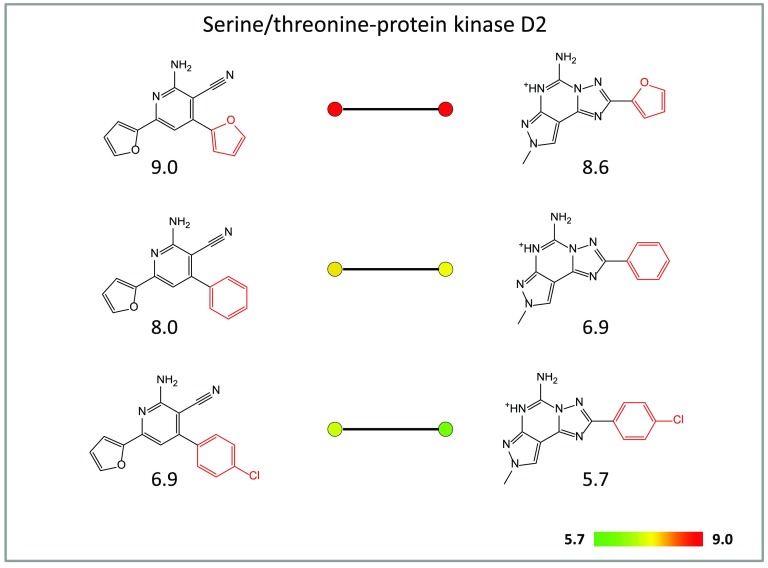
SAR transfer series. An exemplary target-based SAR transfer series is shown. Compound pairs are arranged in the order of increasing potency (from the bottom to the top). Potency progression is monitored by corresponding pairs of color-coded dots using a continuous color spectrum from green (lowest potency value (pK _i_ = 5.7) in the compound data set), over yellow to red (highest potency value; pK _i_ = 9.0). The pK _i_ value of each compound is provided. The core structures are drawn in black and the substituents in red. The compounds are active against serine/threonine-protein kinase D2.

**Figure 3.**
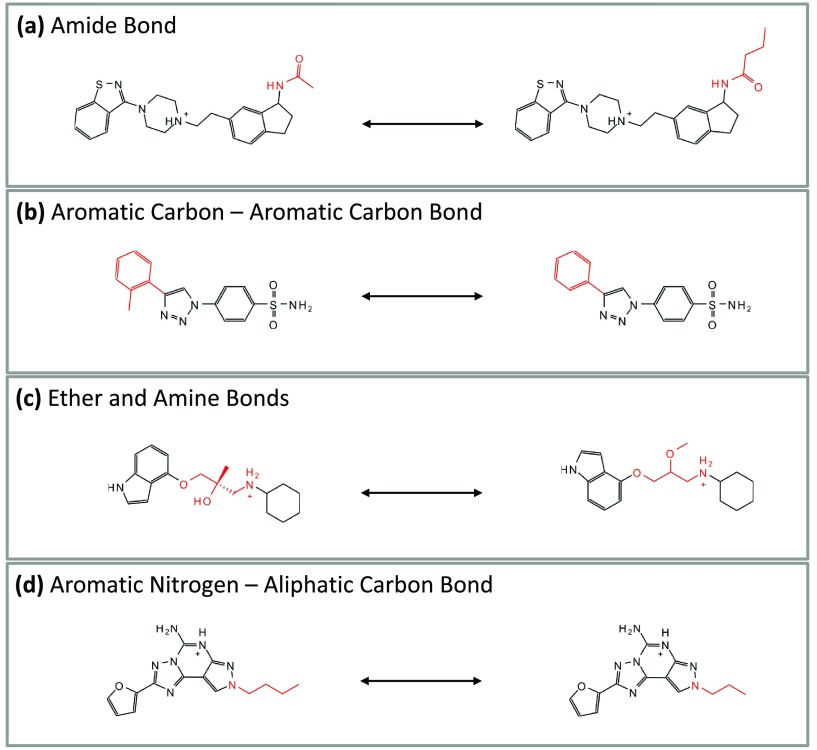
RECAP-MMPs. In ( **a**)–( **d**), four exemplary RECAP-MMPs representing different retrosynthetic rules are shown. For each RECAP-MMP, the chemical transformation is highlighted in red.

**Figure 4.**
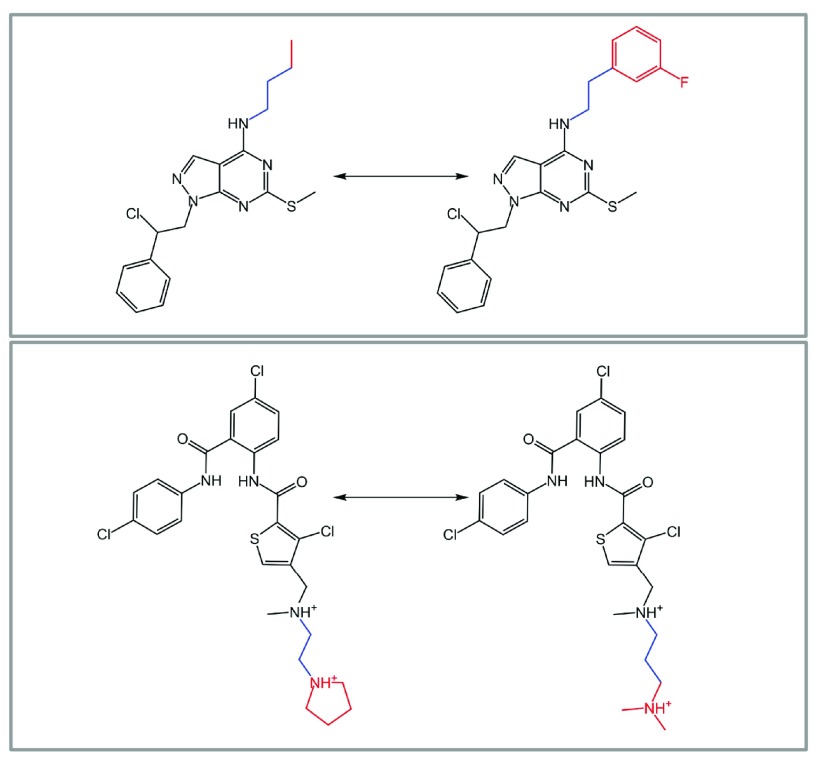
Standard MMPs vs. RECAP-MMPs. Two pairs of compounds that form both standard MMPs and RECAP-MMPs are shown. For each pair, the structural differences between compounds are highlighted. The chemical transformation associated with the standard MMP is colored in red, while the transformation of the RECAP-MMP corresponds to the combination of fragments colored in red and blue.

(1) *Activity cliffs* are generally defined as pairs or groups of compounds that are structurally similar and have large differences in potency ^9– 11^. Accordingly, activity cliffs usually have high SAR information content (because small chemical changes in similar or analogous compounds lead to large potency effects). The assessment of activity cliffs requires clearly defined similarity and potency difference criteria ^9– 11^. The formation of an MMP can be considered as a similarity criterion, which is similarity metric-free and often chemically more intuitive than the use of calculated molecular similarity ^11, 12^. MMP formation as a similarity criterion has led to the introduction of MMP-cliffs ^12^. For MMP-cliffs, a difference in potency of at least two orders of magnitude between cliff-forming compounds was set as a potency difference criterion ^12^. Figure 1 shows exemplary MMP-cliffs.

(2) *SAR transfer* can be rationalized in different ways. For example, a compound series might display similar potency progression against two different targets ^13^. Alternatively, two different compound series with corresponding analogs, i.e., series having different core structures and containing compounds with pairwise corresponding substitutions, might display similar potency progression against a given target ^14^. Such *SAR transfer series* displaying similar target-specific SAR behavior are often sought after in medicinal chemistry as alternative compounds for optimization. Here we focus on these target-based SAR transfer series. Figure 2 shows an example.

(3) Computational generation of MMPs typically involves molecular fragmentation through the systematic deletion of exocyclic single bonds ^5^. Hence, the resulting fragments representing a molecular core and substituent are not derived considering chemical reactions. Accordingly, a transformation relating MMP-forming compounds to each other might not necessarily be interpretable from a synthetic perspective. Hence synthetic accessibility of MMPs might be further improved by considering the reaction information during molecular fragmentation. This has been accomplished by applying the well-known retrosynthetic combinatorial analysis procedure (RECAP) rules ^15^, leading to the introduction of *RECAP-MMPs* ^16^. Representative examples are shown in Figure 3. In addition, examplary differences between standard MMPs and RECAP-MMPs are illustrated in Figure 4.

### MMP generation

For the generation of MMP-cliffs, SAR transfer series, and RECAP-MMPs, transformation size restrictions that limit transformations to meaningful chemical substitutions were introduced ^12^. Specifically, the common core structure had to be at least twice the size of each exchanged substructure. Furthermore, the difference in size of the exchanged fragments was limited to at most eight non-hydrogen atoms and the maximal size of an exchanged fragment was set to 13 non-hydrogen atoms ^12^. Therefore, the largest permitted transformations included, for example, the addition of a substituted ring to a compound or the replacement of a five- or six-membered ring with a substituted condensed two-ring system (with a maximum of 13 atoms). All possible transformation size-restricted MMPs and RECAP-MMPs were calculated using an in-house implementation of the algorithm by Hussain and Rea ^5^ that utilizes the OpenEye toolkit ^17^.

### Compounds and activity data

Compound data were taken from the latest version of ChEMBL (release 17) ^7, 8^. Only compounds with direct interactions (i.e., target relationship type “D”) against human targets at the highest confidence level (target confidence score 9) were selected. Two types of potency measurements were separately considered, i.e., K _i_ (equilibrium constant) and IC _50_ (half-maximal inhibition concentration) values. In order to ensure high data confidence, inactive or inconclusive compounds and compounds with approximate measurements such as “>”, “<”, or “∼” were not considered. For compounds with multiple measurements against the same target, the geometric mean was calculated as the final potency annotation, provided that all values fell within one order of magnitude; otherwise, the compound was discarded. All qualifying compounds were further organized into target sets. A total of 661 and 1203 target sets (consisting of compounds with reported specific activity against a given target) were collected for the K _i_- and IC _50_-based subsets, respectively, as reported in Table 1. The target sets contained a total of 45,353 and 95,685 compounds and 77,421 and 135,291 potency measurements for the K _i_ and IC _50_ subsets, respectively. These target sets provided the basis for the generation of all MMPs.

**Table 1.** Data sets.

Number of	K _i_	IC _50_
**Targets**	661	1203
**Compounds**	45,353	95,685
**Measurements**	77,421	135,291

For the K _i_ and IC _50_ subsets from the latest version of ChEMBL (release 17), the total numbers of targets, compounds, and corresponding potency measurements are reported.

## Results

As a follow-up on the original publications in which MMP-cliffs ^12^, SAR transfer series ^14^, and RECAP-MMPs ^16^ were introduced, all corresponding data sets have been re-generated on the basis of ChEMBL release 17, hence providing up-to-date versions for release. Separate data subsets have been generated for different types of well-defined potency measurements (i.e., assay-dependent IC _50_ vs. assay-independent K _i_ values) to avoid inconsistencies due to simultaneous consideration of different potency measurements that cannot be directly compared.

### MMP-cliffs

Figure 1 illustrates small chemical changes in compound pairs leading to large potency differences that are captured by MMP-cliffs. For ease of structural interpretation, we currently prefer MMP-based activity cliff representations compared to alternative representations that rely on calculated similarity values ^11^. Table 2 provides the MMP-cliff statistics for the current data set. On the basis of K _i_ and IC _50_ measurements, more than 20,000 and 25,000 MMP-cliffs were obtained, respectively, requiring an at least 100-fold difference in potency between cliff-forming compounds. The MMP-cliffs corresponded to ~5% of all MMPs that were generated from ChEMBL compounds with high-confidence activity data. They covered 293 and 500 different targets on the basis of K _i_ and IC _50_ measurements, respectively. In addition to the more conservative potency difference cutoff, MMP-cliffs were also identified when a less stringent criterion was applied, i.e., two compounds forming an MMP were required to have a potency difference of at least one order of magnitude. In this case, as reported in Table 2, nearly 99,000 and more than 126,000 MMP-cliffs were detected in 392 and 726 targets for the K _i_ and IC _50_ subsets, respectively. The proportion of MMP-cliffs increased to approx. 25%.

**Table 2.** MMP and MMP-cliff statistics.

Number of		K _i_	IC _50_
**MMPs**		385,777	537,848
**Targets with MMPs**		467	929
**MMP compounds**		40,454 (89.2%)	80,744 (84.4%)
**∆Potency** **≥ 1 OoM**	**MMP-cliffs**	98,608	126,464
	**% MMP-cliffs**	25.6%	23.5%
	**Targets with MMP-cliffs**	392	726
	**MMP-cliff compounds**	29,976 (66.1%)	50,413 (52.7%)
**∆Potency** **≥ 2 OoM**	**MMP-cliffs**	20,073	25,297
	**% MMP-cliffs**	5.2%	4.7%
	**Targets with MMP-cliffs**	293	500
	**MMP-cliff compounds**	11,760 (25.9%)	16,816 (17.6%)

For the K _i_- and IC _50_-based compound subsets, the number of MMPs, the number of targets for which MMPs were obtained, and the number (and ratio) of compounds that formed MMPs are reported. In addition, the number and proportion of MMP-cliffs derived from all MMPs with potency difference (∆Potency) of at least one order (1 OoM) or two orders of magnitude (2 OoM) are provided, respectively, as well as the number of targets for which MMP-cliffs were obtained and the number (and ratio) of cliff-forming compounds.

### SAR transfer series

SAR transfer series are best rationalised as pairs of compound series active against the same target that have distinct core structures, and consist of corresponding pairs of analogs, as illustrated in Figure 2 for a small series with three pairs. Different from the original analysis of target-based SAR transfer ^14^ that was based upon MMPs without transformation size restrictions, the current analysis has been carried out on the basis of size-restricted MMPs. This modification further supports SAR exploration (because only small chemical changes are considered) and explains a reduction in series numbers compared to the original publication. In Table 3, the numbers of different series available for the current data set are reported. A total of 1270 and 2109 matching series were obtained from the K _i_ and IC _50_ subsets, respectively. Matching series met the structural requirement of consisting of at least three pairs of corresponding analogs. In addition, the potency values of compounds associated with individual series had to span at least two orders of magnitude. From these pre-selected matching series, 157 (K _i_) and 513 (IC _50_) SAR transfer series with at least approximate potency progression and activity against 42 and 54 targets, respectively, were obtained. A subset of 60 (K _i_) and 322 (IC _50_) SAR transfer series displayed strictly corresponding (regular) potency progression (often over different potency ranges) ^14^. These series were active against 23 (K _i_) and 27 (IC _50_) different targets. The size of SAR transfer series with approximate and regular potency progression ranged from three to 12 corresponding pairs of analogs. On average, the SAR transfer series consisted of three to four pairs.

**Table 3.** Target-based SAR transfer series statistics.

Number of	K _i_	IC _50_
**Matching series**	1270	2109
**T_SAR-TS**	157	513
**Targets with T_SAR-TS**	42	54
**T_SAR-TS-RP**	60	322
**Targets with T_SAR-TS-RP**	23	27

For the K _i_ and IC _50_ subsets, the number of qualifying matching compound series is reported. In addition, the number of target-based SAR transfer series with at least approximate potency progression (T_SAR-TS), the subset of SAR transfer series with regular potency progression (T_SAR-TS-RP), and the corresponding numbers of targets are given.

### RECAP-MMPs

The replacement of systematic fragmentation of exocyclic single bonds with a set of 13 retrosynthetic rules for MMP generation reduced the number of MMPs that were obtained by more than half. RECAP-MMP numbers are reported in Table 4. However, (perhaps surprisingly) large numbers of RECAP-MMPs remained for further consideration and assessment of synthetic feasibility. From the K _i_ and IC _50_ subsets, nearly 170,000 and more than 240,000 RECAP-MMPs were obtained with activity against 371 and 778 targets, respectively. Examples are shown in Figure 3.

**Table 4.** RECAP-MMP statistics.

Number of	K _i_	IC _50_
**RECAP-MMPs**	169,889	240,322
**Targets with RECAP-MMPs**	371	778
**RECAP-MMP compounds**	28,529 (62.9%)	53,917 (56.3%)

For the K _i_ and IC _50_ subsets, the number of RECAP-MMPs, the number of targets for which RECAP-MMPs were obtained, and the number (and ratio) of compounds that formed RECAP-MMPs are reported.

## Data availability

All MMP-cliffs, SAR transfer series, and RECAP-MMPs are provided in canonical SMILES representation ^18^ on a per-target basis separately for the K _i_ and IC _50_ subsets. The canonical SMILES representation of compounds was calculated using the Molecular Operating Environment ^19^ on the basis of standardized molecular structures by removing solvents or ions and rebalancing protonation states. Furthermore, the canonical SMILES representation of key fragments (cores) and chemical transformations derived from MMPs and RECAP-MMPs was generated using the OpenEye toolkit ^17^.

ZENODO: Detailed data sets of MMP-cliffs, SAR transfer series, RECAP-MMPs and compound activities, doi: 10.5281/zenodo.8418 ^20^.

## Summary

We have described new and up-to-date MMP-based data sets comprising activity cliffs, SAR transfer series, and second generation retrosynthetic MMPs that have been systematically generated from currently available public domain compounds with high-confidence activity data. Hence, these data sets are comprehensive and have broad target coverage. They are made available without restrictions to the scientific community to aid in SAR analysis, compound design, and other medicinal chemistry applications. It is hoped that these data sets might be of interest and useful to many investigators in this field and catalyse further research efforts.
