# Supplementary material for: Matched molecular pair-based data sets for computer-aided medicinal chemistry
Source: F1000Res. 2014 Feb 21;3:36. Originally published 2014 Feb 4. [Version 2] doi: 10.12688/f1000research.3-36.v2 (PMC3945766; doi:10.12688/f1000research.3-36.v2)
Supplement: Peer review history

# Peer review: Referee response for version 2

**Published:** 2014-02-24

**Authors:** Patrick Walters

**Affiliations:** 1 Vertex Pharmaceuticals Inc., Cambridge, MA, USA

This revised version looks good.

I have read this submission. I believe that I have an appropriate level of expertise to confirm that it is of an acceptable scientific standard.

# Peer review: Referee response for version 1

**Published:** 2014-02-20

**Authors:** Shana Posy

**Affiliations:** 1 Research and Development, Bristol-Myers Squibb, Princeton, NJ, USA

Hu *et al* have compiled a useful set of matched pair datasets based on the CHEMBL database of biological activity. They describe in a straightforward manner the derivation of the datasets and basic concepts relevant for matched pairs. The following suggested modifications to the data provided would enhance their usefulness and completeness:

1. As a result of switching the method for generating MMPs from cleavage of single bonds to a RECAP-based method, the pool of MMPs now includes substitutions of internal fragments (e.g. in Figure 3c) as well as substitution of a terminal R-group (as in Figure 3 examples a, b, and d). Although both types of MMPs involve replacement of a single structural fragment, it may be desirable for many applications to distinguish between core scaffold replacement and R-group variation. It would therefore be helpful to annotate the datasets to easily separate these two classes of MMPs.
   
2. Since the authors filter out IC50s/Kis of indeterminate values, it is unclear how compounds that were clearly inactive were processed. Were compounds with IC50s/Kis that could not be quantified due to a flat dose response curve included in the datasets?
   
3. The authors present a filtered dataset where a number of factors have contributed to rejection of potential MMPs, namely:  the difference in size of the exchanged fragments was limited to 8 heavy atoms; the ratio of the common core fragment to the size of each exchanged fragment had to be>2; and the exchanged fragment could have maximum 13 heavy atoms. While these are reasonable filters to obtain MMPs that truly represent small structural changes, the cutoffs selected are arbitrary and for some targets may exclude MMPs that another user might consider relevant. Rather than providing the final filtered dataset, it would be helpful if the authors would provide the full original datasets with the values of the features used for filtering annotated as extra columns. This would allow maximal flexibility in designing custom MMP sets.
   
4. In the files that list the RECAP MMPs, key fields are missing that would require the user to retrieve the relevant data from CHEMBL in order to perform any analysis: (a) the Target name (only the target CHEMBL ID is provided); and (b) more importantly, the compound activities are not included.
   
5. In the files that list the transfer series, for each matched pair the authors provide the two series cores and full compound smiles, but not the substituted fragments.

I have read this submission. I believe that I have an appropriate level of expertise to confirm that it is of an acceptable scientific standard.

# Peer review: Referee response for version 1

**Published:** 2014-02-18

**Authors:** Patrick Walters

**Affiliations:** 1 Vertex Pharmaceuticals Inc., Cambridge, MA, USA

This paper provides a review of the Bajorath group's recent work on matched molecular pairs (MMP), a technique for exploring structure activity relationships, and identifying chemical transformations that can readily modulate biological activity. The authors focus on recent applications of MMP to large datasets from the publicly accessible ChEMBL database. The paper provides an excellent introduction to those unfamiliar with the MMP technique and with concepts such as activity cliffs. In addition to providing an overview of the recent literature, the authors also provide links to publicly available software and datasets that will provide tutorial materials for those interested in learning more about these powerful techniques. Datasets and software like those described in this paper are valuable resources. A logical next step from this work would be to create interactive tutorials using tools like the iPython Notebook Viewer or knitr.

The presentation is clear, but a few changes may help readers unfamiliar with some of the concepts.

- On p2 the authors refer to " *second generation MMPs*". It would be useful to add a sentence explaining the differences between first and second generation MMPs.
   
- MMP Cliffs which differ in activity by 10-fold may also be interesting. It would be informative to see the number of examples available with a 1 log vs 2 log difference.
   
- In the section (3) on page 3, it would be interesting to provide a specific example of how the results of RECAP generated MMPS differ from those generated using a more "traditional" approach.

This paper provides an excellent gateway to a topic that is becoming increasingly more important in drug discovery. The paper should be of interest to computational and medicinal chemists as well as biologists.

I have read this submission. I believe that I have an appropriate level of expertise to confirm that it is of an acceptable scientific standard.

# Peer review: Referee response for version 1

**Published:** 2014-02-17

**Authors:** Peter Ertl

**Affiliations:** 1 Cheminformatics, Novartis Institutes for Biomedical Research, Basel, Switzerland

The matched molecule pairs approach provides a “chemistry friendly” and intuitive way of expressing relationships among molecules and therefore this manuscript is of importance to all cheminformatics scientists interested in the study of activity cliffs, SAR analysis and in the design of bioactive molecules in general.

The authors extracted several datasets of matched molecule pairs from public databases that could be used as benchmarks in further analysis of this phenomenon. Care has been taken to assure a high quality of data.

The manuscript is well written and the procedure and all results are sufficiently documented and, in addition, all datasets are available for download; therefore I am suggesting only a few minor modifications to the text:

1. Introduction: replace " *the latest release of the ChEMBL*" with the version number.
   
2. Provide a bit more technical information about the in-house implementation of a molecule fragmentation procedure used to generated matched pairs. Was the procedure implemented entirely in-house, or is it based on a publicly available cheminformatics toolkit? (If this is the case, please cite the respective toolkit).
   
3. The authors mention that structures in the download file are available as canonical SMILES. The form of canonical SMILES however will depend on the particular program used to generate it. Please specify whether the original ChEMBL SMILES is included or the canonical SMILES was created by another toolkit

I have read this submission. I believe that I have an appropriate level of expertise to confirm that it is of an acceptable scientific standard.

# Peer review: Referee response for version 1

**Published:** 2014-02-10

**Authors:** Ajay Jain

**Affiliations:** 1 Departments of Biopharmaceutical Sciences and Laboratory Medicine, University of California San Francisco, San Francisco, CA, USA

The data set described by Hu *et al.* is a large set of carefully curated small molecule matched-molecular pairs (MMPs) with high-quality activity data derived from ChEMBL. The set includes examples of structure-activity cliffs, as well as matched SAR-transfer series, both of which are important in the development and validation of activity prediction algorithms. The availability of the MMP data set will be very valuable to researchers that are focused on methods development. The data should also be of interest to those interested in fundamental questions about molecular activity (e.g. questions about the independence and additivity of activity changes that are linked with substituent changes).

I have read this submission. I believe that I have an appropriate level of expertise to confirm that it is of an acceptable scientific standard.
